# Systematic Investigation of the Effect of Powerful Tianma Eucommia Capsule on Ischemic Stroke Using Network Pharmacology

**DOI:** 10.1155/2021/8897313

**Published:** 2021-06-04

**Authors:** Pengcheng Feng, Guixia Li, Yan Huang, Jinhong Pei

**Affiliations:** Department of Basic Medicine, Changzhi Medical College, Changzhi, China

## Abstract

**Background:**

Ischemic stroke (IS) is a serious disease with a high rate of death and disability, and a growing number of people are becoming victims. Existing drugs not only have limited therapeutic effects but also have obvious side effects. Most importantly, drug resistance due to long-term or improper use of drugs is detrimental to patients. Therefore, it is urgent to find some alternative or supplementary medicines to alleviate the current embarrassment. Powerful Tianma Eucommia Capsule (PTEC) is mainly used to treat IS in China for thousands of years; however, the molecular mechanism is not clear.

**Methods:**

Pharmacology ingredients and target genes were filtered and downloaded from websites. A pharmacology ingredient-target gene network was constructed to predict the molecular interactions between ingredients and target genes. Enrichment analysis was performed to explore the possible signal pathways. LeDock was used to simulate the interaction form between proteins and main active ingredients and to deduce key amino acid positions.

**Results:**

Two hundred eighty-nine target genes and seventy-four pharmacological ingredients were obtained from public databases. Several key ingredients (quercetin, kaempferol, and stigmasterol) and primary core target genes (PTGS1, NCOA2, and PRSS1) were detected through ingredient-target gene network analysis. Gene Ontology (GO) and Kyoto Encyclopedia of Genes and Genomes (KEGG) enrichment analysis demonstrated that ingredients affect networks mainly in nuclear receptor activity and *G* protein-coupled amine receptor activity; besides, fluid shear stress and atherosclerosis, human cytomegalovirus infection, and hepatitis B signaling pathways might be the principal therapy ways. A series of presumed key amino acid sites (189ASP, 190SER, 192GLN, 57HIS, and 99TYE) were calculated in PRSS1. Six of the target genes were differentially expressed between male and female patients.

**Conclusions:**

Seven new putative target genes (ACHE, ADRA1A, AR, CHRM3, F7, GABRA1, and PRSS1) were observed in this work. Based on the result of GO and KEGG analysis, this work will be helpful to further demonstrate the molecular mechanism of PTEC treatment of IS.

## 1. Introduction

Ischemic stroke (IS) is a severe disease due to insufficient blood supply or blockage of blood flow to certain parts of the brain. Although IS has a high rate of incidence, recurrence, and disability, as long as the response is fast enough, the harm caused by the disease can be effectively alleviated, elimination of intravascular thrombi within 5 hours [1–3]. Over 1.1 million people are killed every year due to IS, according to the report from World Health Organization (WHO) [4].

Numerous investigations have proved that IS can be cured by removing the thrombus (drugs and thrombectomy), but efficient recanalization remains a challenge [5]. During the clinical treatment of IS, the resistance to certain drugs and side effects have become increasingly obvious [6, 7]. In a study of platelet resistance in juvenile patients with acute IS and its association with early neurological deterioration (END) and recurrent ischemic stroke (RIS), it was found that 24.4% of patients are aspirin resistant, 35.9% are clopidogrel resistant, and 19.2% are both aspirin and clopidogrel resistant [7, 8].

Scientists have done much work to explore the pathogenic mechanisms. Some miRNAs and LncRNAs related to neuronal damage, destruction of blood-brain barrier, inflammation, autophagy, and the occurrence of IS were identified in recent years [9, 10]. Hydrogen may be a complementary therapy to treat IS due to the antioxidative and anti-inflammatory effect [11]. Sonic hedgehog (Shh) signaling pathway and stem cell are related to oxidative stress and neurogenesis. Maybe, they are novel strategies for the treatment of IS [12]. However, researchers are committed to finding therapeutic drugs, including molecular drugs and “new use of old drugs.” Khaksari et al. have shown that the peptide hormone Apelin-13 (serum apolipoprotein-13), originally isolated from the bovine stomach, may protect the blood-brain barrier from ischemic injury through aquaporin, antiapoptotic activity, reduction of cerebral infarction volume, and edema [13]. However, clinical studies had shown that a high level of Apelin-13 was a poor prognostic molecule and often associated with high recurrence rates and high complications [14]. Platelet thrombosis is one of the main reasons for the low cure rate of IS. It has been proven that metformin can inhibit platelet activation and thrombosis, thereby alleviating the condition of IS mice. Xin et al. optimized the derivative biguanide to reduce platelet aggregation and adhesion, and long-term treatment showed no obvious toxicity [15].

Powerful Tianma Eucommia Capsule (PTEC) (a traditional Chinese patent medicine) has good therapeutic effects on dispelling wind and promoting blood circulation, relaxing muscles, and relieving pain. PTEC is mainly used to treat IS clinically, which is made from Tianma (*Gastrodia elata*), Duzhong (*Eucommia ulmoides* Oliver), Zhicaowu, Fuzi (Aconiti Lateralis Radix Praeparata), Duhuo (Radix Angelicae Sinensis), Gaoben (Ligustici Rhizoma et Radix), Xuanshen (Figwort Root), Danggui (Angelicae Sinensis Radix), Dihuang (Rehmanniae Radix), Hujisheng (*Viscum angulatum* Heyne), Qianghuo (Notopterygii Rhizoma et Radix), and Chuanniuxi (Cyathulae Radix) in a specific ratio [16–20]. These traditional Chinese herbal medicines are widely used in the clinical treatment of IS; the therapeutic effect of this drug was far beyond that of a single herbal. Therefore, the ingredients in PTEC may be effective through multiple targets and pathways.

Network pharmacology, a research strategy based on protein-chemical relationships and disease-protein relationships, is used for drug discovery and development and to predict the molecular mechanism of drug treatment of disease [21]. In the study of the treatment mechanism, this technical strategy makes the analysis more comprehensive. In this work, an ingredient-target gene network was constructed to investigate the underlying molecular mechanism of PTEC against IS. Gene Ontology (GO) and Kyoto Encyclopedia of Genes and Genomes (KEGG) enrichment analysis were carried out to investigate the role of target genes in cells and signaling pathways. Molecular docking was performed to verify the interactions between key genes and ingredients. This work was not only helpful to understand the mechanism of cure IS but also meaningful to explore new clinical value not just its traditional applications.

## 2. Materials and Methods

### 2.1. Database Construction

The pharmacological ingredient information of Powerful Tianma Eucommia Capsule was obtained from the TCMSP (https://tcmspw.com/tcmsp.php). The pharmacological ingredients with oral bioavailability (OB ≥ 30%), drug-likeness (DL ≥ 0.18), and drug half-life (HL ≥ 3) were selected as candidates. Related target genes were downloaded from TCMSP according to candidate pharmacological ingredients.

### 2.2. Collection of Ischemic Stroke-Related Proteins

GeneCards (https://www.genecards.org/version5.0) and OMIM (https://omimorg/search/advanced/geneMap) were used to obtain IS-related proteins. “Ischemic stroke, infarction, pectoris, and atherosclerosis” were used as keywords to search in the websites of GeneCards and OMIM. All related proteins were downloaded and summarized.

### 2.3. Construction and Analysis of Networks

An ingredient-target gene network was constructed by Cytoscape 3.7.1. Each independent gene and effective ingredient of the medicine is called a node, and the line emitted by each node is called a degree. The degree values were evaluated and exported by Cytoscape option network analyzer. In this work, the nodes, with a higher degree than the average value, were considered to play critical roles in the treatment of IS. STRING (version 11.0, https://string-db.org/) was used to calculate and predict protein-protein interaction (PPI) network. To have high confidence, the interaction parameter was set at 0.9. Cytoscape 3.7.1 was applied to visualize two networks. The top 50 genes were shown by the MCC algorithm in CytoHubba.

### 2.4. Enrichment Analysis of Target Genes

GO enrichment analysis was applied to interpret the biological function. Kyoto Encycolopedia of Genes and Genomes (KEGG) enrichment analysis was used to elaborate the pathways of the target genes by *R* packages (colorspace, stringi, DOSE, clusterProfiler, and pathview). The two-side hypergeometric test method was used in this part. The measure is *p* value less than 0.05.

### 2.5. Molecular Docking

The 3D structure of ingredients and crystal structures of proteins was downloaded from TCMSP and Protein Data Bank, separately [22]. LePro was adopted to process protein receptor files and Chimera 1.13.1 used was to minimize ligand structure. All parameters were set to default values. LeDock (http://www.lephar.com/) was applied to molecular dock. LeDock, based on simulated annealing and evolutionary optimization, is more accurate and efficient than some commercial programs [23]. The interactions among main ingredients and the target genes were visualized and displayed in 3D diagrams by PyMOL 1.8 and Chimera 1.13.1. Amino acids, 3 Å far from ligand, were shown in a 3D structure diagram. And H-bond was predicted between ostensive amino acids and ligands.

### 2.6. Determining Gene Expression Atlas

The Human Protein Atlas (https://www.proteinatlas.org) was an important resource that can provide single gene expression patterns in tissue, cell line, pathology, brain, and blood. The gene expression patterns of ten genes (NCOA2, PTGS1, PRSS1, ESR1, F7, RELA, CTNNB1, FOS, NCOA1, and NR3C1) were downloaded and summarized.

### 2.7. Statistical Analysis

In this work, *R* language (version 3.6.3) was used to perform statistical analysis. All values are expressed as mean ± standard deviation. *p* value < 0.05 was considered statistically significant. In the part of GO and KEGG enrichment analysis, *Q* value and *p* adjusted value were both less than 0.05, and this value was considered statistically significant.

## 3. Result

### 3.1. Collection of Pharmacological Ingredients and Target Genes

The designations of all Chinese herbal medicines from PTEC were used as keywords to search for pharmacological ingredients from CTMSP. Seventy-four ingredients were filtered by parameters of OB, DL, and HL. Two hundred eighty-nine protein targets were found according to 74 pharmacological ingredients. Perl was used to translate protein names into gene symbols. The proteins that did not match the gene symbol were deleted. Finally, a hundred four gene names were translated into gene symbols. Two hundred eighty-nine ingredient-target gene relationships were confirmed. The detailed information is shown in Tables S1 and S2.

### 3.2. Screening of Common Genes

**Figure 1:**
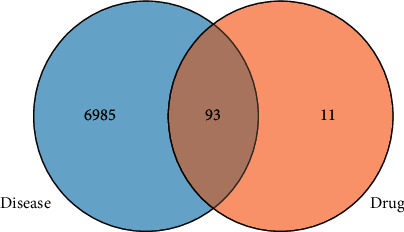
The collection of common genes. The blue section represents the proteins obtained from disease-relevant websites; the orange section represents the proteins that were the targets of PTEC; the common section (brown part) represents proteins that are not only disease-related but also drug targets.

Seven thousand forty-four genes were obtained from the GeneCards website that provides annotated human genes information. Fifty-five genes were obtained from OMIM. A total of 7078 unique genes were collected. Ninety-three common proteins were filtered by the Venn tool. According to 289 ingredient-target relationships and the gene symbols of 93 proteins, 41 pharmacological ingredients were obtained. The relationship of proteins is shown in Figure 1.

### 3.3. Construction of Ingredient-Target Network

**Figure 2:**
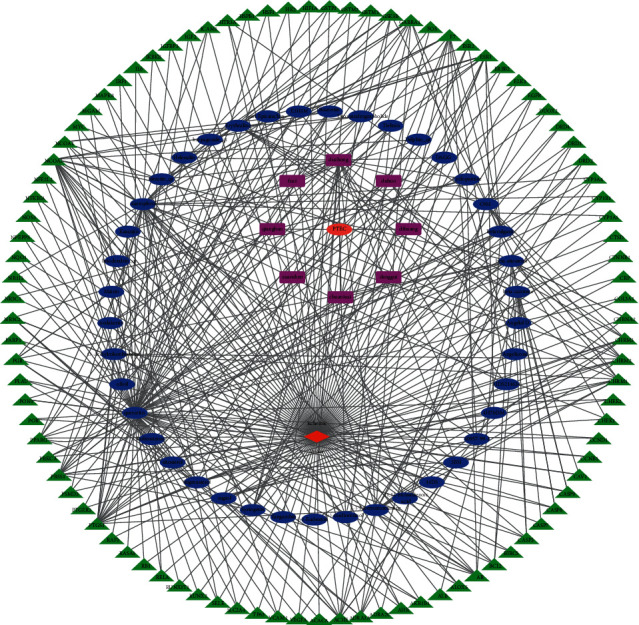
Ingredient-target network. Green section: target genes; blue section: pharmacological ingredients; purple section: Chinese traditional herbs; red section: IS; orange section: PTEC. The edges between two nodes mean that they have a subordinate relationship or can interact with each other.

The herbs, Tianma, Zhiwucao, Gaoben, and Hujisheng, without qualified pharmacological ingredients, were deleted, so the pharmacological ingredients were without IS-related target genes. The ingredient-target network consisted of 41 pharmacological ingredients, 93 target genes, 8 herbs, 1 drug, and 1 disease. This network was composed of 144 nodes and 412 edges. The ingredient-target network is shown in Figure 2. In this network, target genes, such as NCOA2, PTGS1, PRSS1, F7, and ESR1, were considered critical target genes (degree ≥ 10) regulated by pharmacological ingredients. Additionally, quercetin, kaempferol, *β*-sitosterol, stigmasterol, (-)-tabernemontanine, cinchonan-9-al, 6'-methoxy-, (9R)-, *β*-carotene, and *β*-vulgarin were predicted as the major pharmacological ingredients. Sitosterol is a compound contained in Dihuang, Hujisheng, and Qianghuo. *β*-sitosterol is a unique ingredient in Hujisheng and Chuanniuxi. Quercetin and kaempferol consist of 71 and 31 disease target genes, respectively, of which 26 are common targets. Five common targets were found among *β*-sitosterol, quercetin, and kaempferol. A single ingredient could regulate multiple target genes, whereas a single gene could be regulated by multiple ingredients. This network was composed of multiple ingredients and multiple target genes. Taken together, these data demonstrated that “multicomponent multitarget gene” was one of the mechanisms of PTEC treatment of IS.

### 3.4. Obtaining Pivotal Target Genes

**Figure 3:**
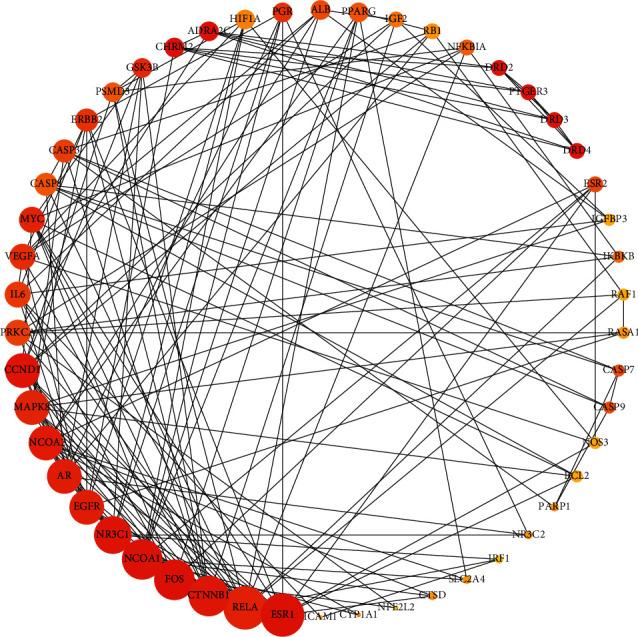
The exploration of PPI with 93 target genes using STRING. The area of the circle is proportional to the size of the degree.

All 93 genes were uploaded to STRING to explore the protein-protein interaction network. The gene pairs that can interact with each other were downloaded in CSV file. The interaction relationship of the top 50 genes was shown by the MCC algorithm in CytoHubba in Figure 3. The degree of each protein was reflected by the size of the circle. RESR1, RELA, CTNNB1, FOS, and NCOA1 might play important roles in this network (degree ≥ 10).

### 3.5. GO and KEGG Enrichment Analysis

**Figure 4:**
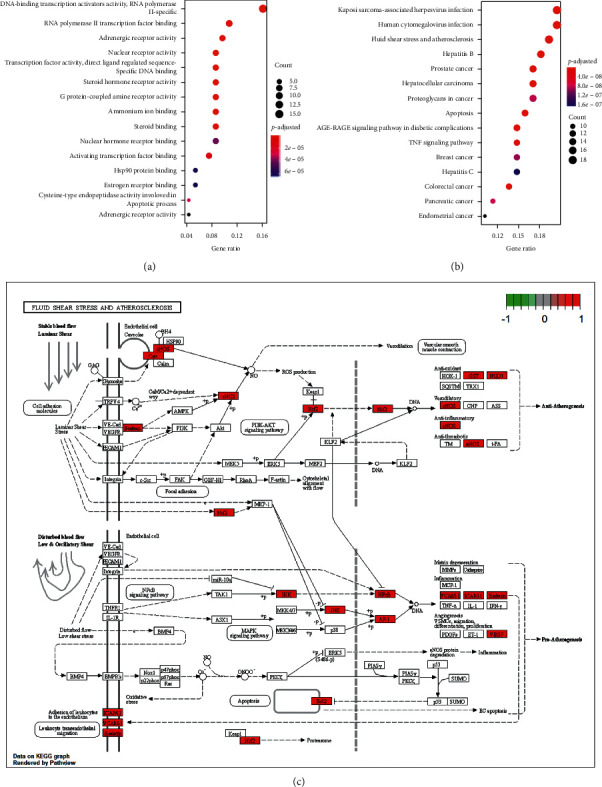
Function enrichment analysis. In Figures 4(a) and 4(b), the abscissa represents the proportion of genes enriched in the pathway among all 93 target genes, and the ordinate shows the name of the terms that were enriched. The size of dot represents the proportion of genes. From red to blue, the significance of GO enrichment gradually decreased. (a) TOP 15 GO enrichment analysis. (b) TOP 15 pathways enrichment analysis. (c) Fluid shear stress and atherosclerosis signaling pathway.

GO enrichment was performed by *R* 3.6.3 to elucidate the multiple biological functions of 93 genes and the top 15 enrichment terms are shown in Figure 4(a). The GO categorization indicated that putative targets were mainly enriched in *G* protein-coupled amine receptor activity, activating transcription factor binding, DNA-binding transcription activator activity, RNA polymerase II-specific, cysteine-type endopeptidase activity involved in apoptotic process, neurotransmitter receptor activity, ammonium ion binding, and so on. The detailed information of 114 GO enrichment terms is shown in Table S3.

KEGG pathway enrichment analysis was conducted and 120 pathways were filtered by *p* adjusted value (less than 0.05): multiple cancers signaling pathways (prostate cancer, colorectal cancer, pancreatic cancer, endometrial cancer, breast cancer, and gastric cancer), fluid shear stress and atherosclerosis, TNF signaling pathway, VEGF signaling pathway, cAMP signaling pathway, and so on. The detailed information of KEGG enrichment analysis is shown in Table S4. IS is a kind of senile disease attributed to vascular sclerosis. Target genes were enriched in fluid shear stress and atherosclerosis signaling pathway was marked in red (Figure 4(c)).

### 3.6. Molecular Docking Simulation

**Figure 5:**
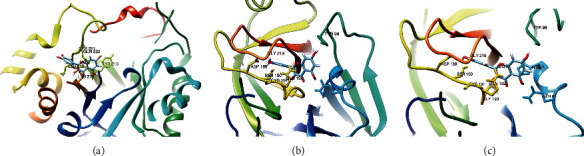
Molecular docking of PRSS1, PTGS1, and their ligands. (a) PTGS1 and quercetin; (b) PRSS1 and quercetin; (c) PRSS1 and kaempferol. The 3D structures of amino acids within 3 Å of the ligand were shown. Hydrogen bonds between ligand and amino acid were marked in blue.

In this work, LeDock was used to predict the pose of ligand and key amino acid in target genes. The target genes and ingredients with a degree greater than 10 were used to dock. In this part, twelve target genes and eleven ingredients were selected to the molecular dock. At the same time, NCOA2 (PDB ID: 5KRH, ligand: 6WN) and PRSS1 (PDB: 1FXY, ligand: 0G6) were analyzed as control. The docking affinity of NCOA2 and 6WN was −6.82 kcal/mol. The predicted pose and location of the two ligands were almost the same as the native, and concrete results were supplied in Figures S1 and S2. Thirty-eight molecular docking jobs were performed on LeDock and the docking affinity scores are shown in Table S5. Molecular docking between quercetin and numinous target genes is shown in Figure 5. Affinity scores of molecular dockings between quercetin and PRSS1, PTGS1, and NCOA1 were −7.09, −6.8, and −6.58 kcal/mol, respectively; these data indicated that great binding interactions were existing between quercetin and these genes. The hydrogen bonds between ligands and amino acids (191CYS and 193 GLY) may be vital in ligands binding to PRSS1. The worth of 189ASP, 190SER, 192GLN, 57HIS, and 99TYE could not be ignored.

### 3.7. Determining Gene Expression Atlas

**Figure 6:**
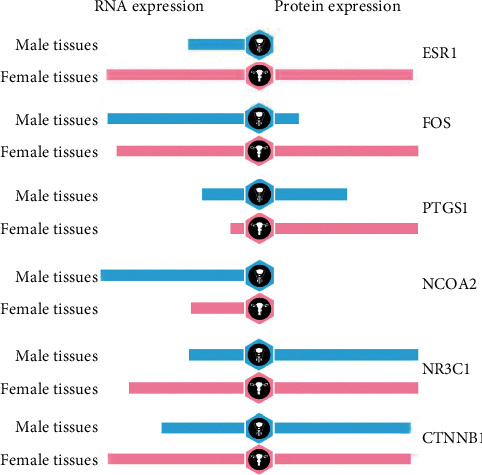
Details of the expression of six target genes in gonads and sexual organs. The bar chart on the left represents the expression level of RNA, and the right represents the expression level of protein. Blue means the tissues from males, while pink means the tissues from females.

Thanks to the Human Protein Atlas, the expressions of 10 genes have been shown. PTGS1 was highly expressed in brain and subsets of cells in tissue stroma and megakaryocytes. PRSS1, ESR1, and NR3C1 were highly expressed in immune cells. No difference in expressions of F7, NCOA1, PRSS1, and RELA was found among sexual organs and gonads between males and females. Six genes' expressions in sexual organs are shown in Figure 6.

## 4. Discussion

Cerebrovascular disease is one of the world's leading causes of death and disability, even in developed countries with more advanced medical equipment and higher medical standards [24, 25]. IS, especially in the elderly, is becoming one of the most common cerebrovascular diseases. Thrombolytic agents, anticoagulants, and antiplatelet agents are the mainstream drugs; some limitations were observed in timeliness and effectiveness of their application (prime time for thrombolysis is within 4.5 h and urgent anticoagulation may lead to intracranial bleeding) [26]. In the East, Chinese herbal medicine has been used to treat stroke for thousands of years due to its cheap and easily available resources. A variety of Chinese patent medicines can be used to treat IS, and many scholars have conducted research on medicines such as Tribulus Terrestris [27], Shuxuening injection [28, 29], baicalin [30], geniposide [31], Chuanxiong-Chishao [32], Musk [33], Nao An capsule [34], Deng-Zhan-Xi-Xin injection [35], scutellarin [36], and Qingkailing injection [37].

In this work, we explored the potential mechanism of PTEC against IS. Seventy-four ingredients were screened from PTEC. Quercetin as a main ingredient that possessed the potential to treat IS was not only found in this work [34], but previous studies have shown that quercetin cures IS by inhibiting *μ*-calpain, Na^+^ channel, and acid-sensing ion channels [38, 39]. NAD^+^-dependent deacetylases (SIRT), a gene family, are related to many diseases including IS, and quercetin could alleviate the condition of stroke patients by activating SIRT1 [40]. Quercetin protected against neuronal injury by inhibiting the activation of MMP-9 and *µ*-calpain and attenuating blood-brain barrier disruption [38]. Acid-sensing ion channels (ASICs) were relevant to lots of diseases; quercetin could deactivate some related proteins [41]. Some benefits for IS patients were obtained by kaempferol inhibition of mitochondrial apoptotic pathway [42, 43]. In summary, the results of previous studies had shown that quercetin and kaempferol could treat or effectively alleviate the condition of IS patients. In this study, PTEC contained these two components. Therefore, we believed that PTEC could treat IS with a scientific basis [39, 44].

Some of the main target genes (ESR1 and RELA) were also found from Precursors works [29, 34]. An association between variation in the estrogen receptor-alpha gene (ESR1) and cerebrovascular disease is related to gender [45]. PTGS1 was highly expressed in females than males. Some diversities in single nucleotide polymorphisms (SNP) of the ESR1 have different effects on the risk of IS, such as the onset age and the probability of developing cerebrovascular disease [46]. RELA is a part of the nuclear factor Kappa-B family (NF-*κ*B), which is concerned with inflammation and occurrence and progression of cancer. RELA-specific acetylation is critical to the prevention and treatment of IS [47, 48]. The genetic variation in PTGS1 may increase the risk for cerebrovascular disease events [49]. Abnormally expressed NCOA2 is usually associated with signal transduction and disease progression and is a potential drug target for many cancers [50, 51]. The polymorphisms and activation of coagulation factor VII gene (F7) may increase the risk of IS in adult patients [52–54]. These studies confirmed that PTGS1, NCOA2, PRSS1, F7, and ESR1 are involved in the occurrence, development, and deterioration of IS. PTEC may achieve therapeutic effects by regulating these genes. Furthermore, ACHE, ADRA1A, AR, CHRM3, F7, GABRA1, and PRSS1 were not previously found; they might be the new targets against IS.

Thanks to GO and KEGG, numerous inflammation signaling pathways and target therapeutic pathways were discovered. PTGS1 was referred to as antioxidant activity (GO: 0016209), heme binding (GO: 0020037), tetrapyrrole binding (GO: 0046906), and peroxidase activity (GO: 0004601) during GO enrichment analysis. NCOA2 was referred to as nuclear hormone receptor binding (GO: 0035257), hormone receptor binding (GO: 0051427), nuclear receptor transcription coactivator activity (GO: 0030374), and transcription coactivator activity (GO: 0003713). PRSS1 and F7 were responsible for endopeptidase activity (GO: 0004175). ESR1 was related to nuclear receptor activity (GO: 0004879), steroid hormone receptor activity (GO: 0003707), and steroid binding (GO: 0005496). Several target genes were enriched in immune- and inflammation-related pathways (JAK-STAT signaling pathway, NF-*κ*B signaling pathway, Toll-like receptor signaling pathway, B cell receptor signaling pathway, and T cell receptor signaling pathway). NF-*κ*B signaling pathway, MAPK signaling pathway, and HIF-1 signaling pathway were found to be associated with IS by immense amounts of research. Fifty genes were enriched in the MAPK signaling pathway, including FOS and RELA. IS patients may be worsening, when MAPK signaling was activated [55]. TNF-*α* could activate glial cells and lead to damage of the blood-brain barriers [56].

Six of the target genes (ESR1, FOS, PTGS1, NR3C1, NCOA2, and CTNNB1) were differentially expressed between males and females. One hundred twenty-three differentially expressed genes were identified from GSE22255 (Gene Expression Omnibus data) [57]. It was necessary to classify some medicines to males and females separately. These six genes highly expressed in females indicated that PTEC may more efficient for females.

Obviously, some limitations were observed when the results were just predicted by bioinformatic methods. It was necessary to perform cell experiments or animal experiments to confirm the predicted mechanism. What is more, the hypothesis that PTEC was more efficient for females needs to be verified.

## Data Availability

The data used in this study can be found on the public website.

## Abbreviations

IS: Ischemic stoke
PTEC: Powerful Tianma Eucommia Capsule
END: Early neurological deterioration
RIS: Recurrent ischemic stroke
Shh: Sonic hedgehog
TCMSP: Traditional Chinese medicine system pharmacology database and analysis platform
OMIM: Online Mendelian Inheritance in Man
GO/KEGG: Gene Ontology/Kyoto Encyclopedia of Genes and Genomes
OB/DL/HL: Oral bioavailability/drug-likeness/drug half-life
PPI: Protein-protein interaction.

## References

[1] GBD 2016 Brain and Other CNS Cancer Collaborators (2019). Global, regional, and national burden of brain and other CNS cancer, 1990–2016: a systematic analysis for the global burden of disease study 2016. *The Lancet Neurology*.

[2] GBD 2016 Stroke Collaborators (2019). Global, regional, and national burden of stroke, 1990–2016: a systematic analysis for the global burden of disease study 2016. *The Lancet Neurology*.

[3] Benmoyal-Segal L. (2004). Acetylcholinesterase/paraoxonase interactions increase the risk of insecticide-induced Parkinson’s disease. *The FASEB Journal*.

[4] Chen Z., Jiang B., Ru X. (2017). Mortality of stroke and its subtypes in China: results from a nationwide population-based survey. *Neuroepidemiology*.

[5] Denorme F., Langhauser F., Desender L. (2016). ADAMTS13-mediated thrombolysis of t-PA-resistant occlusions in ischemic stroke in mice. *Blood*.

[6] Musumeci L., Kuijpers M. J., Gilio K. (2015). Dual-specificity phosphatase 3 deficiency or inhibition limits platelet activation and arterial thrombosis. *Circulation*.

[7] Yi X., Wang C., Liu P., Fu C., Lin J., Chen Y. (2016). Antiplatelet drug resistance is associated with early neurological deterioration in acute minor ischemic stroke in the Chinese population. *Journal of Neurology*.

[8] Yi X., Lin J., Wang Y. (2016). Association of cytochrome P450 genetic variants with clopidogrel resistance and outcomes in acute ischemic stroke. *Journal of Atherosclerosis and Thrombosis*.

[9] Howard G., Banach M., Cushman M. (2015). Is blood pressure control for stroke prevention the correct goal?. *Stroke*.

[10] Ren W., Yang X. (2018). Pathophysiology of long non-coding RNAs in ischemic stroke. *Frontiers in Molecular Neuroence*.

[11] Li H., Yin L., Pengfei Y., Liu J. (2018). Hydrogen as a complementary therapy against ischemic stroke: a review of the evidence. *Journal of the Neurological Sciences*.

[12] Xu W., Zheng J., Gao L., Li T., Zhang J., Shao A. (2017). Neuroprotective effects of stem cells in ischemic stroke. *Stem Cells International*.

[13] Chu H., Yang X., Huang C., Gao Z., Tang Y., Dong Q. (2017). Apelin-13 protects against ischemic blood-brain barrier damage through the effects of aquaporin-4. *Cerebrovascular Diseases*.

[14] Xie F., Colantonio L. D., Curtis J. R. (2016). Linkage of a population-based cohort with primary data collection to medicare claims. *American Journal of Epidemiology*.

[15] Xin G., Ming Y., Ji C. (2020). Novel potent antiplatelet thrombotic agent derived from biguanide for ischemic stroke. *European Journal of Medicinal Chemistry*.

[16] Hong L.-Z., Gu W.-w., Ni Y. (2013). Postischemic long-term treatment with qiangli tianma duzhong capsule improves brain functional recovery via the improvement of hemorrheology and the inhibition of platelet aggregation in a rat model of focal cerebral ischemia. *Evidence-Based Complementary and Alternative Medicine*.

[17] Gao L., Wang P.-P., Liu Q. (2008). Removing phlegm and dispelling stasis method combined with western medicine for treatment of cerebrovascular stenosis. *Zhongguo Zhong Xi Yi Jie He Za Zhi Zhongguo Zhongxiyi Jiehe Zazhi = Chinese Journal of Integrated Traditional and Western Medicine/Zhongguo Zhong Xi Yi Jie He Xue Hui, Zhongguo Zhong Yi Yan Jiu Yuan Zhu Ban*.

[18] Shen X., Wang G., Zhao Y. (2006). Clinical analysis of qiangli tianma duzhong capsule in treating chronic cerebral insufficiency. *ChineseTraditional Patent Medicine*.

[19] Han L., Wang J. (2006). Vague categorization of the metal elements in Chinese patent medicines for treating cerebrovascular diseases. *Chinese Journal of Clinical Rehabilitation*.

[20] Jin J. (2009). Protective effect study of qiangli tianma duzhong capsule on focal cerebral ischemia rats. *Journal of Liaoning University of TCM*.

[21] Shao L. (2002). Advances in TCM symptomatology of rheumatoid arthritis. *Journal of Traditional Chinese Medicine*.

[22] Thanki N., Feng Z., Marvin J. (2000). The protein data bank. *Nuclc Acids Research*.

[23] Wang Z., Sun H., Yao X. (2016). Comprehensive evaluation of ten docking programs on a diverse set of protein-ligand complexes: the prediction accuracy of sampling power and scoring power. *Physical Chemistry Chemical Physics*.

[24] Pivina L. M., Moldagalieva Z. T., Muzdubayeva Z. E., Belikhina T. I., Markabayeva A. M., Zhunussova T. (2015). Medical and social problem of cardiovascular diseases in Kazakhstan. *Science and Healthcare*.

[25] Ma X. M., Liu M., Liu Y. Y., Ma L. L, Jiang Y, Chen X. H (2016). Ischemic preconditioning protects against ischemic brain injury. *Neural Regeneration Research*.

[26] Bansal S., Sangha K. S., Khatri P. (2013). Drug treatment of acute ischemic stroke. *American Journal of Cardiovascular Drugs*.

[27] Wang Y., Guo W., Liu Y. (2019). Investigating the protective effect of gross saponins of tribulus terrestris fruit against ischemic stroke in rat using metabolomics and network pharmacology. *Metabolites*.

[28] Ming L., Ying C., Tiechan Z. (2018). Tnfrsf12a-mediated atherosclerosis signaling and inflammatory response as a common protection mechanism of shuxuening injection against both myocardial and cerebral ischemia-reperfusion injuries. *Frontiers in Pharmacology*.

[29] Cui Q., Zhang Y.-L., Ma Y.-H. (2020). A network pharmacology approach to investigate the mechanism of shuxuening injection in the treatment of ischemic stroke. *Journal of Ethnopharmacology*.

[30] Xu T., Ma C., Fan S. (2018). Systematic understanding of the mechanism of baicalin against ischemic stroke through a network pharmacology approach. *Evidence-Based Complementary and Alternative Medicine*.

[31] Zhou Y.-X., Zhang R.-Q., Rahman K., Cao Z.-X., Zhang H., Peng C. (2019). Diverse pharmacological activities and potential medicinal benefits of geniposide. *Evidence-Based Complementary and Alternative Medicine*.

[32] Wang Y., Guo G., Yang B.-r. (2017). Synergistic effects of chuanxiong-chishao herb-pair on promoting angiogenesis at network pharmacological and pharmacodynamic levels. *Chinese Journal of Integrative Medicine*.

[33] Zhang C., Liao Y., Liu L. (2020). A network pharmacology approach to investigate the active compounds and mechanisms of musk for ischemic stroke. *Evidence-Based Complementary and Alternative Medicine*.

[34] Chen C., Li H.-L., Yi Y., Fan H.-J., Chen C. (2019). Network pharmacology-based study on the active substances and mechanism of nao an capsule in treatment of ischemic stroke. *European Journal of Integrative Medicine*.

[35] Zhao J., Lv C., Wu Q. (2019). Computational systems pharmacology reveals an antiplatelet and neuroprotective mechanism of deng-zhan-xi-xin injection in the treatment of ischemic stroke. *Pharmacological Research*.

[36] Meng Z. Q., Wu J. R., Zhu Y. L. (2020). Revealing the common mechanisms of scutellarin in angina pectoris and ischemic stroke treatment via a network pharmacology approach. *Chinese Journal of Integrative Medicine*.

[37] Ma C., Wang X., Xu T. (2020). An integrative pharmacology-based analysis of refined qingkailing injection against cerebral ischemic stroke: a novel combination of baicalin, geniposide, cholic acid, and hyodeoxycholic acid. *Frontiers in Pharmacology*.

[38] Kumar Pandey A., Pandey S. C., Bhattacharya P., Patnaik R. (2016). A possible therapeutic potential of quercetin through inhibition of *μ*-calpain in hypoxia induced neuronal injury: a molecular dynamics simulation study. *Neural Regeneration Research*.

[39] Patel R. V., Mistry B. M., Shinde S. K., Syed R., Singh V., Shin H.-S. (2018). Therapeutic potential of quercetin as a cardiovascular agent. *European Journal of Medicinal Chemistry*.

[40] Bai X., Yao L., Ma X., Xu X. (2018). Small molecules as SIRT modulators. *Mini-Reviews in Medicinal Chemistry*.

[41] Mukhopadhyay M., Singh A., Sachchidanand S., Bera A. K. (2017). Quercetin inhibits acid-sensing ion channels through a putative binding site in the central vestibular region. *Neuroscience*.

[42] Wu B., Luo H., Zhou X. (2017). Succinate-induced neuronal mitochondrial fission and hexokinase II malfunction in ischemic stroke: therapeutical effects of kaempferol. *Biochimica et Biophysica Acta (BBA)—Molecular Basis of Disease*.

[43] Zhou Y. P., Li G. C. (2020). Kaempferol protects cell damage in in vitro ischemia reperfusion model in rat neuronal PC12 cells. *Biochimica et Biophysica Acta (BBA)—Molecular Basis of Disease*.

[44] Bijou E. A., William H., Charles H. D., Bardien S., Ekpo O. E. (201s8). Rutin as a potent antioxidant: implications for neurodegenerative disorders. *Oxidative Medicine Cellular Longevity*.

[45] Markoula S., Milionis H., Lazaros L. (2012). Associations of ESR2 aluI (G/A) polymorphism with ischemic stroke in caucasians. *Journal of the Neurological Ences*.

[46] Lazaros L., Markoula S., Xita N. (2010). Association of estrogen receptor-alpha gene polymorphisms with stroke risk in patients with metabolic syndrome. *Acta Neurologica Scandinavica*.

[47] Xie W., Zhu T., Dong X. (2019). HMGB1-triggered inflammation inhibition of notoginseng leaf triterpenes against cerebral ischemia and reperfusion injury via MAPK and NF-*κ*B signaling pathways. *Biomolecules*.

[48] Lanzillotta A., Pignataro G., Branca C. (2012). Targeted acetylation of NF-kappaB/RelA and histones by epigenetic drugs reduces post-ischemic brain injury in mice with an extended therapeutic window. *Neurobiology of Disease*.

[49] Lee C. R., North K. E., Bray M. S., Couper D. J., Heiss G., Zeldin D. C. (2007). Cyclooxygenase polymorphisms and risk of cardiovascular events: the atherosclerosis risk in communities (ARIC) study. *Clinical Pharmacology & Therapeutics*.

[50] Yu J., Wu W. K. K., Liang Q. (2015). Disruption of NCOA2 by recurrent fusion with LACTB2 in colorectal cancer. *Oncogene*.

[51] Silva M. P., Barros-Silva J. D., Vieira J. (2016). NCOA2 is a candidate target gene of 8q gain associated with clinically aggressive prostate cancer. *Genes, Chromosomes and Cancer*.

[52] Lopaciuk S., Windyga J., Watala C. W. (2010). Polymorphisms in the factor VII gene and ischemic stroke in young adults. *Blood Coagulation & Fibrinolysis*.

[53] Vries P. S. d, Sabater-Lleal M., Huffman J. E. (2019). A genome-wide association study identifies new loci for factor vii and implicates factor vii in ischemic stroke etiology. *Blood*.

[54] Zakai N. A., Lange L., Longstreth W. T. (2011). Association of coagulation-related and inflammation-related genes and factor VIIc levels with stroke: the cardiovascular health study. *Journal of Thrombosis and Haemostasis*.

[55] Maddahi A., Kruse L. S., Chen Q.-W., Edvinsson L. (2011). The role of tumor necrosis factor-*α* and TNF-*α* receptors in cerebral arteries following cerebral ischemia in rat. *Journal of Neuroinflammation*.

[56] Tuttolomondo A., Pecoraro R., Pinto A. (2014). Studies of selective TNF inhibitors in the treatment of brain injury from stroke and trauma: a review of the evidence to date. *Drug Design, Development and Therapy*.

[57] Zhu W., Nan Y., Wang S., Liu W. (2019). Bioinformatics analysis of gene expression profiles of sex differences in ischemic stroke. *BioMed Research International*.

