# Supplementary material for: Systematic Investigation of the Effect of Powerful Tianma Eucommia Capsule on Ischemic Stroke Using Network Pharmacology
Source: Evid Based Complement Alternat Med. 2021 Jun 4;2021:8897313. doi: 10.1155/2021/8897313 (PMC8203382; doi:10.1155/2021/8897313)
Supplement: Supplementary Materials — All tables and molecular docking control lines can be found in supplementary materials. [file 8897313.f1.zip › 8897313.f1/supplement figures.docx]

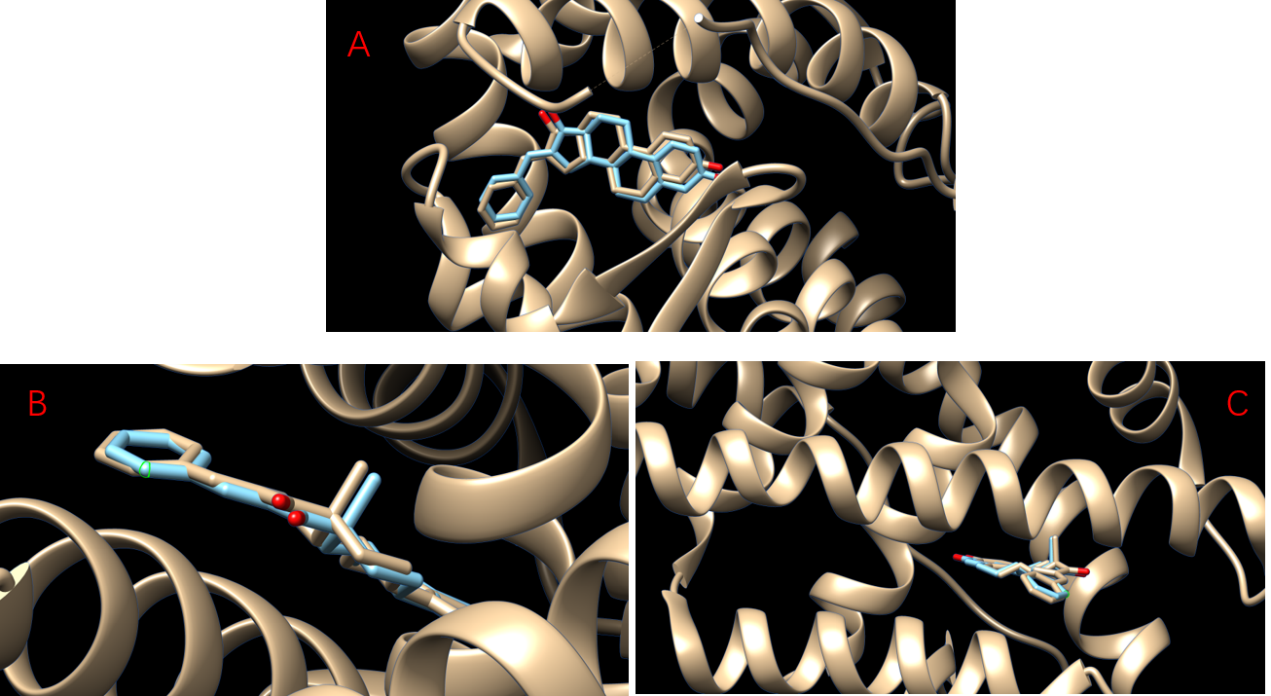


**Figure S1** molecular docking of NCOA2 and 6WN. NCOA2 and native ligand were marked gold, predicted ligand marked blue; A, B and C were taken photos from different angle


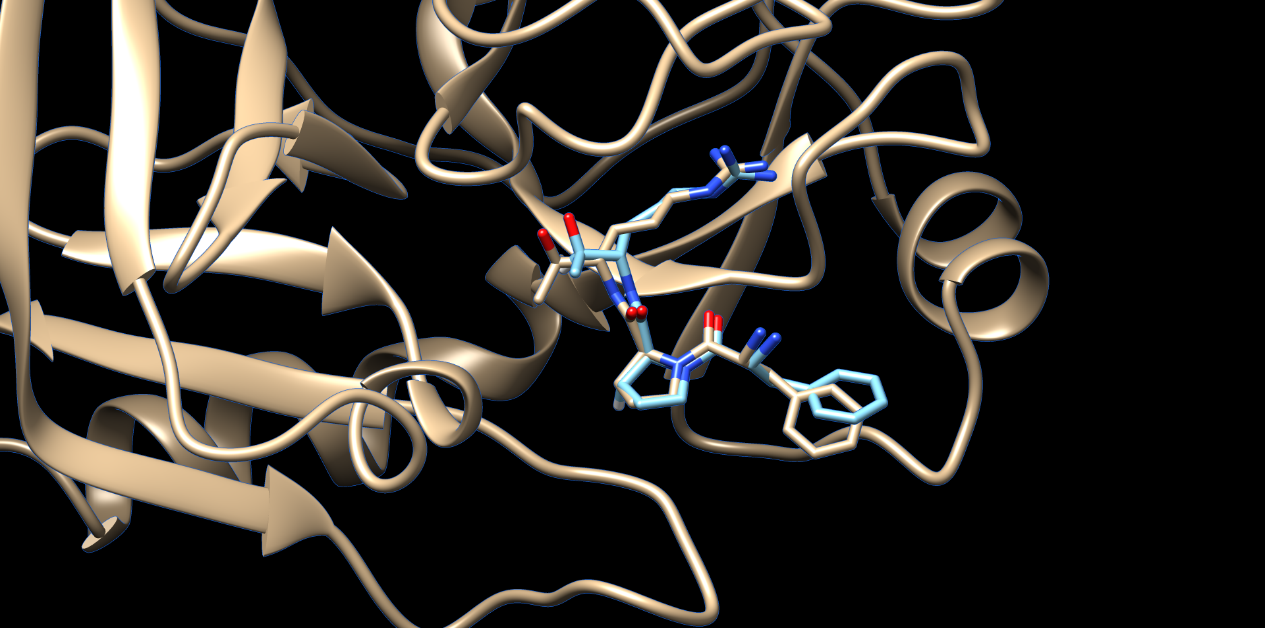


**Figure S2** molecular docking of PRSS1 and 0G6. PRSS1 and native ligand were marked gold, predicted ligand marked blue
