# Supplementary material for: Systematic Investigation of the Effect of Powerful Tianma Eucommia Capsule on Ischemic Stroke Using Network Pharmacology
Source: Evid Based Complement Alternat Med. 2021 Jun 4;2021:8897313. doi: 10.1155/2021/8897313 (PMC8203382; doi:10.1155/2021/8897313)
Supplement: Supplementary Materials — All tables and molecular docking control lines can be found in supplementary materials. [file 8897313.f1.zip › 8897313.f1/Table 1.docx]

Table 1 the detail information of Pharmacological ingredients

| Mol ID | CAS | OB (%) | DL | HL |
| --- | --- | --- | --- | --- |
| MOL001925 | 23180-57-6 | 68.18 | 0.4 | 8.81 |
| MOL002222 | 511-05-7 | 36.11 | 0.28 | 14.62 |
| MOL007657 | N | 38.63 | 0.77 | 12.18 |
| MOL007658 | N | 62.57 | 0.42 | 5.16 |
| MOL007659 | 148000-43-5 | 36.62 | 0.4 | 16.34 |
| MOL007660 | N | 33.17 | 0.82 | 12.27 |
| MOL007662 | N | 122.87 | 0.32 | 10.55 |
| MOL000359 | 83-46-5 | 36.91 | 0.75 | 5.37 |
| MOL001951 | 7380-40-7 | 41.73 | 0.42 | 2.06 |
| MOL002881 | 520-34-3 | 31.14 | 0.27 | 16.34 |
| MOL004792 | 495-31-8 | 57.12 | 0.69 | 7.16 |
| MOL011962 | 131623-14-8 | 32.02 | 0.67 | 12.9 |
| MOL011968 | N | 33.07 | 0.78 | 2.19 |
| MOL011971 | N | 67.57 | 0.31 | 3.7 |
| MOL011975 | N | 62.97 | 0.48 | 0.77 |
| MOL000338 | 446-71-9 | 51.61 | 0.27 | 16.05 |
| MOL000351 | 552-54-5 | 47.14 | 0.34 | 13.54 |
| MOL000354 | 480-19-3 | 49.6 | 0.31 | 14.34 |
| MOL000538 | 6900-87-4 | 31.39 | 0.26 | 19.87 |
| MOL002211 | 2091-39-6 | 39.99 | 0.2 | 5.6 |
| MOL002388 | 17670-06-3 | 57.76 | 0.28 | 1.43 |
| MOL002392 | 19662-71-6 | 46.69 | 0.37 | 7.7 |
| MOL002393 | N | 34.52 | 0.18 | 26.05 |
| MOL002394 | N | 34.52 | 0.18 | 26.75 |
| MOL002395 | 79233-15-1 | 56.3 | 0.31 | 2.79 |
| MOL002397 | 39089-30-0 | 51.73 | 0.73 | 11.1 |
| MOL002398 | 521-88-0 | 69.56 | 0.34 | 13.15 |
| MOL002401 | N | 43.1 | 0.85 | 12.05 |
| MOL002406 | N | 39.43 | 0.38 | 10.95 |
| MOL002410 | 198126-85-1 | 34.06 | 0.53 | 15.72 |
| MOL002415 | N | 51.87 | 0.66 | 13.14 |
| MOL002416 | 3175-95-9 | 30.96 | 0.24 | 22.64 |
| MOL002419 | 106032-53-5 | 82.54 | 0.21 | 3.85 |
| MOL002421 | 1357-76-2 | 84.08 | 0.25 | 28.93 |
| MOL002422 | 7633-68-3 | 50.82 | 0.73 | 11.59 |
| MOL002423 | 16298-90-1 | 33.41 | 0.19 | 26.06 |
| MOL002433 | N | 41.52 | 0.22 | 10.51 |
| MOL002434 | N | 38.16 | 0.8 | 7 |
| MOL000073 | 35323-91-2 | 48.96 | 0.24 | 0.63 |
| MOL000211 | 472-15-1 | 55.38 | 0.78 | 8.87 |
| MOL000422 | 520-18-3 | 41.88 | 0.24 | 14.74 |
| MOL000443 | 466-77-3 | 49.18 | 0.55 | 11.11 |
| MOL002058 | 40957-99-1 | 57.2 | 0.62 | 2.04 |
| MOL002773 | 7235-40-7 | 37.18 | 0.58 | 4.36 |
| MOL004367 | 2955-23-9 | 62.23 | 0.41 | 2.27 |
| MOL005922 | 7374-79-0 | 43.35 | 0.77 | 2.94 |
| MOL006709 | N | 92.43 | 0.55 | 1.83 |
| MOL007059 | N | 32.16 | 0.41 | 22.51 |
| MOL007563 | 13060-14-5 | 57.53 | 0.81 | 3.61 |
| MOL008240 | N | 56.32 | 0.36 | 4.18 |
| MOL009007 | 99633-12-2 | 30.51 | 0.85 | 4.5 |
| MOL009009 | 40957-99-1 | 87.19 | 0.62 | 1.39 |
| MOL009015 | 2134-98-7 | 58.67 | 0.61 | 19.73 |
| MOL009027 | 4449-51-8 | 55.42 | 0.82 | 14.67 |
| MOL009029 | N | 51.44 | 0.4 | 7.54 |
| MOL009030 | N | 30.1 | 0.24 | 2.2 |
| MOL009031 | 572-59-8 | 68.22 | 0.4 | 3.94 |
| MOL009038 | 29307-60-6 | 45.58 | 0.83 | 10.25 |
| MOL009042 | 6754-13-8 | 77.01 | 0.19 | 4.34 |
| MOL009047 | 526-06-7 | 33.29 | 0.62 | 2.24 |
| MOL009053 | 4263-87-0 | 50.76 | 0.39 | 7.58 |
| MOL009055 | 32221-58-2 | 49.81 | 0.37 | 1.4 |
| MOL009057 | 573-44-4 | 53.14 | 0.8 | 3.78 |
| MOL011604 | 4423-37-4 | 36.82 | 0.37 | 14.74 |
| MOL003608 | 23180-65-6 | 60.04 | 0.26 | 3.71 |
| MOL004777 | 83199-39-7 | 34.85 | 0.34 | 0.72 |
| MOL004778 | N | 46.03 | 0.34 | 1.77 |
| MOL004780 | N | 30.99 | 0.19 | 15.93 |
| MOL004782 | N | 45.19 | 0.34 | 2.14 |
| MOL000449 | 83-48-7 | 43.83 | 0.76 | 5.57 |
| MOL000098 | 73123-10-1 | 46.43 | 0.28 | 14.4 |
| MOL000358 | 83-46-5 | 36.91 | 0.75 | 5.36 |
| MOL012286 | 51068-94-1 | 68.75 | 0.39 | 19.96 |
| MOL012298 | 19466-41-2 | 32.69 | 0.47 | 3.92 |
