# Supplementary material for: Systematic Investigation of the Effect of Powerful Tianma Eucommia Capsule on Ischemic Stroke Using Network Pharmacology
Source: Evid Based Complement Alternat Med. 2021 Jun 4;2021:8897313. doi: 10.1155/2021/8897313 (PMC8203382; doi:10.1155/2021/8897313)
Supplement: Supplementary Materials — All tables and molecular docking control lines can be found in supplementary materials. [file 8897313.f1.zip › 8897313.f1/Table 2.docx]

Table 2 The targets information of Pharmacological ingredients

| MolId | Degree of mollecular | Symbol | Degree of gene |
| --- | --- | --- | --- |
| MOL000098 | 72 | ACACA | 2 |
| MOL000098 | 72 | ACHE | 10 |
| MOL000422 | 32 | ACHE | 10 |
| MOL009015 | 13 | ACHE | 10 |
| MOL002222 | 7 | ACHE | 10 |
| MOL009029 | 6 | ACHE | 10 |
| MOL007059 | 6 | ACHE | 10 |
| MOL009053 | 4 | ACHE | 10 |
| MOL002392 | 4 | ACHE | 10 |
| MOL004792 | 3 | ACHE | 10 |
| MOL000358 | 16 | ADRA1A | 7 |
| MOL000449 | 13 | ADRA1A | 7 |
| MOL009015 | 13 | ADRA1A | 7 |
| MOL009031 | 12 | ADRA1A | 7 |
| MOL002222 | 7 | ADRA1A | 7 |
| MOL007059 | 6 | ADRA1A | 7 |
| MOL009031 | 12 | ADRA2C | 2 |
| MOL000098 | 72 | AHR | 3 |
| MOL000422 | 32 | AHR | 3 |
| MOL000098 | 72 | AHSA1 | #N/A |
| MOL000422 | 32 | AHSA1 | #N/A |
| MOL000098 | 72 | AKR1B1 | 3 |
| MOL000449 | 13 | AKR1B1 | 3 |
| MOL000422 | 32 | AKR1C3 | #N/A |
| MOL002773 | 12 | ALB | 2 |
| MOL000098 | 72 | ALOX5 | 3 |
| MOL000422 | 32 | ALOX5 | 3 |
| MOL000098 | 72 | AR | 8 |
| MOL000422 | 32 | AR | 8 |
| MOL009015 | 13 | AR | 8 |
| MOL012286 | 10 | AR | 8 |
| MOL011604 | 9 | AR | 8 |
| MOL003608 | 7 | AR | 8 |
| MOL009027 | 4 | AR | 8 |
| MOL000098 | 72 | BCL2 | 5 |
| MOL000422 | 32 | BCL2 | 5 |
| MOL000358 | 16 | BCL2 | 5 |
| MOL002773 | 12 | BCL2 | 5 |
| MOL000098 | 72 | BIRC5 | 2 |
| MOL000098 | 72 | CASP3 | 5 |
| MOL000422 | 32 | CASP3 | 5 |
| MOL000358 | 16 | CASP3 | 5 |
| MOL002773 | 12 | CASP3 | 5 |
| MOL002773 | 12 | CASP7 | 2 |
| MOL000098 | 72 | CASP8 | 4 |
| MOL000358 | 16 | CASP8 | 4 |
| MOL002773 | 12 | CASP8 | 4 |
| MOL000098 | 72 | CASP9 | 4 |
| MOL000358 | 16 | CASP9 | 4 |
| MOL002773 | 12 | CASP9 | 4 |
| MOL000098 | 72 | CAV1 | 3 |
| MOL002773 | 12 | CAV1 | 3 |
| MOL000098 | 72 | CCNB1 | 2 |
| MOL000098 | 72 | CCND1 | 2 |
| MOL012286 | 10 | CHEK1 | 3 |
| MOL002398 | 4 | CHEK1 | 3 |
| MOL000098 | 72 | CHEK2 | 2 |
| MOL000422 | 32 | CHRM1 | 10 |
| MOL000358 | 16 | CHRM1 | 10 |
| MOL000449 | 13 | CHRM1 | 10 |
| MOL009015 | 13 | CHRM1 | 10 |
| MOL009031 | 12 | CHRM1 | 10 |
| MOL000443 | 7 | CHRM1 | 10 |
| MOL003608 | 7 | CHRM1 | 10 |
| MOL002222 | 7 | CHRM1 | 10 |
| MOL007059 | 6 | CHRM1 | 10 |
| MOL000422 | 32 | CHRM2 | 9 |
| MOL000358 | 16 | CHRM2 | 9 |
| MOL000449 | 13 | CHRM2 | 9 |
| MOL009015 | 13 | CHRM2 | 9 |
| MOL009031 | 12 | CHRM2 | 9 |
| MOL000443 | 7 | CHRM2 | 9 |
| MOL003608 | 7 | CHRM2 | 9 |
| MOL002222 | 7 | CHRM2 | 9 |
| MOL000358 | 16 | CHRM3 | 8 |
| MOL000449 | 13 | CHRM3 | 8 |
| MOL009015 | 13 | CHRM3 | 8 |
| MOL009031 | 12 | CHRM3 | 8 |
| MOL000443 | 7 | CHRM3 | 8 |
| MOL002222 | 7 | CHRM3 | 8 |
| MOL009047 | 3 | CHRM3 | 8 |
| MOL000358 | 16 | CHRM4 | #N/A |
| MOL009015 | 13 | CHRM4 | #N/A |
| MOL009031 | 12 | CHRM4 | #N/A |
| MOL000443 | 7 | CHRM4 | #N/A |
| MOL002222 | 7 | CHRM4 | #N/A |
| MOL009015 | 13 | CHRM5 | #N/A |
| MOL009031 | 12 | CHRM5 | #N/A |
| MOL000443 | 7 | CHRM5 | #N/A |
| MOL002222 | 7 | CHRM5 | #N/A |
| MOL000358 | 16 | CHRNA2 | 4 |
| MOL009015 | 13 | CHRNA2 | 4 |
| MOL000443 | 7 | CHRNA2 | 4 |
| MOL000098 | 72 | COL3A1 | 2 |
| MOL000098 | 72 | CRP | 2 |
| MOL002773 | 12 | CTNNB1 | 2 |
| MOL000449 | 13 | CTRB1 | #N/A |
| MOL000098 | 72 | CTSD | 2 |
| MOL000098 | 72 | CYP1A1 | 3 |
| MOL000422 | 32 | CYP1A1 | 3 |
| MOL000098 | 72 | CYP1B1 | 3 |
| MOL000422 | 32 | CYP1B1 | 3 |
| MOL000098 | 72 | CYP3A4 | 4 |
| MOL000422 | 32 | CYP3A4 | 4 |
| MOL002773 | 12 | CYP3A4 | 4 |
| MOL000098 | 72 | DCAF5 | #N/A |
| MOL000098 | 72 | DIO1 | #N/A |
| MOL000422 | 32 | DIO1 | #N/A |
| MOL009015 | 13 | DRD2 | 4 |
| MOL009031 | 12 | DRD2 | 4 |
| MOL002222 | 7 | DRD2 | 4 |
| MOL009015 | 13 | DRD3 | 3 |
| MOL009031 | 12 | DRD3 | 3 |
| MOL009031 | 12 | DRD4 | 2 |
| MOL000098 | 72 | DUOX2 | 2 |
| MOL000098 | 72 | EGFR | 3 |
| MOL009031 | 12 | EGFR | 3 |
| MOL000098 | 72 | EIF6 | #N/A |
| MOL000098 | 72 | ELK1 | 2 |
| MOL000098 | 72 | ERBB2 | 2 |
| MOL000098 | 72 | ERBB3 | #N/A |
| MOL012286 | 10 | ESR1 | 11 |
| MOL011604 | 9 | ESR1 | 11 |
| MOL003608 | 7 | ESR1 | 11 |
| MOL009029 | 6 | ESR1 | 11 |
| MOL008240 | 6 | ESR1 | 11 |
| MOL009053 | 4 | ESR1 | 11 |
| MOL002398 | 4 | ESR1 | 11 |
| MOL007662 | 4 | ESR1 | 11 |
| MOL004792 | 3 | ESR1 | 11 |
| MOL000073 | 3 | ESR1 | 11 |
| MOL012286 | 10 | ESR2 | 4 |
| MOL011604 | 9 | ESR2 | 4 |
| MOL009055 | 6 | ESR2 | 4 |
| MOL000098 | 72 | F7 | 11 |
| MOL000422 | 32 | F7 | 11 |
| MOL012286 | 10 | F7 | 11 |
| MOL011604 | 9 | F7 | 11 |
| MOL009029 | 6 | F7 | 11 |
| MOL009055 | 6 | F7 | 11 |
| MOL008240 | 6 | F7 | 11 |
| MOL002058 | 4 | F7 | 11 |
| MOL006709 | 3 | F7 | 11 |
| MOL009009 | 3 | F7 | 11 |
| MOL000098 | 72 | FOS | 2 |
| MOL000098 | 72 | GABRA1 | 10 |
| MOL000422 | 32 | GABRA1 | 10 |
| MOL000358 | 16 | GABRA1 | 10 |
| MOL000449 | 13 | GABRA1 | 10 |
| MOL009015 | 13 | GABRA1 | 10 |
| MOL000443 | 7 | GABRA1 | 10 |
| MOL003608 | 7 | GABRA1 | 10 |
| MOL004780 | 3 | GABRA1 | 10 |
| MOL009042 | 2 | GABRA1 | 10 |
| MOL012286 | 10 | GSK3B | 6 |
| MOL011604 | 9 | GSK3B | 6 |
| MOL009055 | 6 | GSK3B | 6 |
| MOL008240 | 6 | GSK3B | 6 |
| MOL007662 | 4 | GSK3B | 6 |
| MOL000098 | 72 | GSTM1 | 3 |
| MOL000422 | 32 | GSTM1 | 3 |
| MOL000098 | 72 | GSTM2 | 3 |
| MOL000422 | 32 | GSTM2 | 3 |
| MOL000098 | 72 | GSTP1 | 3 |
| MOL000422 | 32 | GSTP1 | 3 |
| MOL000098 | 72 | HIF1A | 2 |
| MOL000098 | 72 | HK2 | 2 |
| MOL000098 | 72 | HSF1 | 2 |
| MOL000098 | 72 | HSPB1 | 2 |
| MOL009015 | 13 | HTR3A | 3 |
| MOL009031 | 12 | HTR3A | 3 |
| MOL000098 | 72 | ICAM1 | 3 |
| MOL000422 | 32 | ICAM1 | 3 |
| MOL000098 | 72 | IGF2 | 2 |
| MOL000098 | 72 | IGFBP3 | 2 |
| MOL000422 | 32 | IKBKB | 2 |
| MOL000098 | 72 | IL6 | 2 |
| MOL000098 | 72 | IRF1 | 2 |
| MOL000422 | 32 | MAPK8 | 2 |
| MOL000098 | 72 | MGAM | 2 |
| MOL000098 | 72 | MYC | 3 |
| MOL002773 | 12 | MYC | 3 |
| MOL000449 | 13 | NCOA1 | 6 |
| MOL007059 | 6 | NCOA1 | 6 |
| MOL002881 | 5 | NCOA1 | 6 |
| MOL002395 | 4 | NCOA1 | 6 |
| MOL004777 | 2 | NCOA1 | 6 |
| MOL000098 | 72 | NCOA2 | 19 |
| MOL000422 | 32 | NCOA2 | 19 |
| MOL000358 | 16 | NCOA2 | 19 |
| MOL000449 | 13 | NCOA2 | 19 |
| MOL011604 | 9 | NCOA2 | 19 |
| MOL009055 | 6 | NCOA2 | 19 |
| MOL002881 | 5 | NCOA2 | 19 |
| MOL000359 | 4 | NCOA2 | 19 |
| MOL002058 | 4 | NCOA2 | 19 |
| MOL002395 | 4 | NCOA2 | 19 |
| MOL009047 | 3 | NCOA2 | 19 |
| MOL006709 | 3 | NCOA2 | 19 |
| MOL009009 | 3 | NCOA2 | 19 |
| MOL002388 | 3 | NCOA2 | 19 |
| MOL009057 | 2 | NCOA2 | 19 |
| MOL004367 | 2 | NCOA2 | 19 |
| MOL007563 | 2 | NCOA2 | 19 |
| MOL002211 | 2 | NCOA2 | 19 |
| MOL000098 | 72 | NFE2L2 | 2 |
| MOL000098 | 72 | NFKBIA | 2 |
| MOL000098 | 72 | NOS3 | 2 |
| MOL000098 | 72 | NPEPPS | 2 |
| MOL000098 | 72 | NQO1 | 2 |
| MOL000098 | 72 | NR1I3 | 3 |
| MOL000422 | 32 | NR1I3 | 3 |
| MOL009027 | 4 | NR3C1 | 2 |
| MOL000449 | 13 | NR3C2 | 6 |
| MOL000359 | 4 | NR3C2 | 6 |
| MOL009027 | 4 | NR3C2 | 6 |
| MOL012298 | 2 | NR3C2 | 6 |
| MOL007658 | 2 | NR3C2 | 6 |
| MOL000098 | 72 | PARP1 | 2 |
| MOL009015 | 13 | PDE10A | #N/A |
| MOL000422 | 32 | PGR | 7 |
| MOL000358 | 16 | PGR | 7 |
| MOL000449 | 13 | PGR | 7 |
| MOL000359 | 4 | PGR | 7 |
| MOL002395 | 4 | PGR | 7 |
| MOL000211 | 2 | PGR | 7 |
| MOL000098 | 72 | PLAU | 3 |
| MOL000449 | 13 | PLAU | 3 |
| MOL000098 | 72 | PON1 | 3 |
| MOL000358 | 16 | PON1 | 3 |
| MOL000098 | 72 | POR | 2 |
| MOL000098 | 72 | PPARG | 8 |
| MOL000098 | 72 | PPARG | 8 |
| MOL000422 | 32 | PPARG | 8 |
| MOL000422 | 32 | PPARG | 8 |
| MOL012286 | 10 | PPARG | 8 |
| MOL011604 | 9 | PPARG | 8 |
| MOL008240 | 6 | PPARG | 8 |
| MOL000098 | 72 | PRKCA | 3 |
| MOL000358 | 16 | PRKCA | 3 |
| MOL000098 | 72 | PRSS1 | 12 |
| MOL000422 | 32 | PRSS1 | 12 |
| MOL012286 | 10 | PRSS1 | 12 |
| MOL011604 | 9 | PRSS1 | 12 |
| MOL009029 | 6 | PRSS1 | 12 |
| MOL008240 | 6 | PRSS1 | 12 |
| MOL007059 | 6 | PRSS1 | 12 |
| MOL002881 | 5 | PRSS1 | 12 |
| MOL009053 | 4 | PRSS1 | 12 |
| MOL002392 | 4 | PRSS1 | 12 |
| MOL007662 | 4 | PRSS1 | 12 |
| MOL000098 | 72 | PSMD3 | 3 |
| MOL000422 | 32 | PSMD3 | 3 |
| MOL000098 | 72 | PTGER3 | 2 |
| MOL000098 | 72 | PTGS1 | 19 |
| MOL000422 | 32 | PTGS1 | 19 |
| MOL000358 | 16 | PTGS1 | 19 |
| MOL000449 | 13 | PTGS1 | 19 |
| MOL009015 | 13 | PTGS1 | 19 |
| MOL009031 | 12 | PTGS1 | 19 |
| MOL012286 | 10 | PTGS1 | 19 |
| MOL000443 | 7 | PTGS1 | 19 |
| MOL003608 | 7 | PTGS1 | 19 |
| MOL009029 | 6 | PTGS1 | 19 |
| MOL009055 | 6 | PTGS1 | 19 |
| MOL002881 | 5 | PTGS1 | 19 |
| MOL002058 | 4 | PTGS1 | 19 |
| MOL002392 | 4 | PTGS1 | 19 |
| MOL002398 | 4 | PTGS1 | 19 |
| MOL000073 | 3 | PTGS1 | 19 |
| MOL002388 | 3 | PTGS1 | 19 |
| MOL004780 | 3 | PTGS1 | 19 |
| MOL000098 | 72 | RAF1 | 2 |
| MOL000098 | 72 | RASA1 | 2 |
| MOL000098 | 72 | RASSF1 | #N/A |
| MOL000098 | 72 | RB1 | 2 |
| MOL000098 | 72 | RELA | 3 |
| MOL000422 | 32 | RELA | 3 |
| MOL000098 | 72 | RUNX1T1 | 2 |
| MOL000098 | 72 | RUNX2 | 2 |
| MOL000098 | 72 | SELE | 3 |
| MOL000422 | 32 | SELE | 3 |
| MOL000098 | 72 | SLC2A4 | 3 |
| MOL000422 | 32 | SLC2A4 | 3 |
| MOL000098 | 72 | TP63 | 2 |
| MOL000098 | 72 | VCAM1 | 3 |
| MOL000422 | 32 | VCAM1 | 3 |
| MOL000098 | 72 | VEGFA | 3 |
| MOL002773 | 12 | VEGFA | 3 |
