# Supplementary material for: Systematic Investigation of the Effect of Powerful Tianma Eucommia Capsule on Ischemic Stroke Using Network Pharmacology
Source: Evid Based Complement Alternat Med. 2021 Jun 4;2021:8897313. doi: 10.1155/2021/8897313 (PMC8203382; doi:10.1155/2021/8897313)
Supplement: Supplementary Materials — All tables and molecular docking control lines can be found in supplementary materials. [file 8897313.f1.zip › 8897313.f1/Table 3.docx]

Table 3 GO enrichment information

| ID | Description | GeneRatio | p.adjust |
| --- | --- | --- | --- |
| GO:0004879 | nuclear receptor activity | 8/93 | 2.08E-08 |
| GO:0098531 | transcription factor activity, direct ligand regulated sequence-specific DNA binding | 8/93 | 2.08E-08 |
| GO:0003707 | steroid hormone receptor activity | 8/93 | 6.03E-08 |
| GO:0008227 | G protein-coupled amine receptor activity | 8/93 | 6.97E-08 |
| GO:0070405 | ammonium ion binding | 8/93 | 3.96E-07 |
| GO:0001085 | RNA polymerase II transcription factor binding | 10/93 | 4.55E-07 |
| GO:0001228 | DNA-binding transcription activator activity, RNA polymerase II-specific | 15/93 | 4.55E-07 |
| GO:0030594 | neurotransmitter receptor activity | 9/93 | 4.89E-07 |
| GO:0005496 | steroid binding | 8/93 | 1.46E-06 |
| GO:0033613 | activating transcription factor binding | 7/93 | 1.14E-05 |
| GO:0097153 | cysteine-type endopeptidase activity involved in apoptotic process | 4/93 | 3.11E-05 |
| GO:0035257 | nuclear hormone receptor binding | 8/93 | 4.13E-05 |
| GO:0004935 | adrenergic receptor activity | 4/93 | 4.54E-05 |
| GO:0051879 | Hsp90 protein binding | 5/93 | 6.07E-05 |
| GO:0030331 | estrogen receptor binding | 5/93 | 6.4E-05 |
| GO:1901338 | catecholamine binding | 4/93 | 7.43E-05 |
| GO:0044389 | ubiquitin-like protein ligase binding | 10/93 | 0.000107 |
| GO:0051427 | hormone receptor binding | 8/93 | 0.00011 |
| GO:0015464 | acetylcholine receptor activity | 4/93 | 0.00011 |
| GO:0016209 | antioxidant activity | 6/93 | 0.00011 |
| GO:0035258 | steroid hormone receptor binding | 6/93 | 0.000155 |
| GO:0042165 | neurotransmitter binding | 5/93 | 0.000169 |
| GO:0042562 | hormone binding | 6/93 | 0.00025 |
| GO:0004952 | dopamine neurotransmitter receptor activity | 3/93 | 0.00025 |
| GO:0031625 | ubiquitin protein ligase binding | 9/93 | 0.000317 |
| GO:0043295 | glutathione binding | 3/93 | 0.000317 |
| GO:0004993 | G protein-coupled serotonin receptor activity | 4/93 | 0.000377 |
| GO:0099589 | serotonin receptor activity | 4/93 | 0.000377 |
| GO:1900750 | oligopeptide binding | 3/93 | 0.000377 |
| GO:0070491 | repressing transcription factor binding | 5/93 | 0.000429 |
| GO:0019825 | oxygen binding | 4/93 | 0.000429 |
