# Supplementary material for: Systematic Investigation of the Effect of Powerful Tianma Eucommia Capsule on Ischemic Stroke Using Network Pharmacology
Source: Evid Based Complement Alternat Med. 2021 Jun 4;2021:8897313. doi: 10.1155/2021/8897313 (PMC8203382; doi:10.1155/2021/8897313)
Supplement: Supplementary Materials — All tables and molecular docking control lines can be found in supplementary materials. [file 8897313.f1.zip › 8897313.f1/Table 4.docx]

Table 4 KEGG enrichment information

| ID | Description | GeneRatio | p.adjust |
| --- | --- | --- | --- |
| hsa05215 | Prostate cancer | 15/88 | 9.54E-12 |
| hsa05418 | Fluid shear stress and atherosclerosis | 17/88 | 9.54E-12 |
| hsa05167 | Kaposi sarcoma-associated herpesvirus infection | 18/88 | 8.82E-11 |
| hsa05161 | Hepatitis B | 16/88 | 7.83E-10 |
| hsa05163 | Human cytomegalovirus infection | 18/88 | 1.03E-09 |
| hsa04933 | AGE-RAGE signaling pathway in diabetic complications | 13/88 | 1.54E-09 |
| hsa05210 | Colorectal cancer | 12/88 | 3.35E-09 |
| hsa04210 | Apoptosis | 14/88 | 4.42E-09 |
| hsa04668 | TNF signaling pathway | 13/88 | 4.42E-09 |
| hsa05225 | Hepatocellular carcinoma | 15/88 | 6.17E-09 |
| hsa05205 | Proteoglycans in cancer | 15/88 | 9.07E-08 |
| hsa05224 | Breast cancer | 13/88 | 1E-07 |
| hsa05212 | Pancreatic cancer | 10/88 | 1.29E-07 |
| hsa05213 | Endometrial cancer | 9/88 | 1.58E-07 |
| hsa05160 | Hepatitis C | 13/88 | 1.65E-07 |
| hsa05162 | Measles | 12/88 | 4.01E-07 |
| hsa05169 | Epstein-Barr virus infection | 14/88 | 4.06E-07 |
| hsa04215 | Apoptosis - multiple species | 7/88 | 4.81E-07 |
| hsa04917 | Prolactin signaling pathway | 9/88 | 6.41E-07 |
| hsa04919 | Thyroid hormone signaling pathway | 11/88 | 7.41E-07 |
| hsa01524 | Platinum drug resistance | 9/88 | 8.04E-07 |
| hsa04115 | p53 signaling pathway | 9/88 | 8.04E-07 |
| hsa01522 | Endocrine resistance | 10/88 | 8.82E-07 |
| hsa05134 | Legionellosis | 8/88 | 1.44E-06 |
| hsa04151 | PI3K-Akt signaling pathway | 17/88 | 1.81E-06 |
| hsa05219 | Bladder cancer | 7/88 | 2.05E-06 |
| hsa04066 | HIF-1 signaling pathway | 10/88 | 2.05E-06 |
| hsa04915 | Estrogen signaling pathway | 11/88 | 2.05E-06 |
| hsa05170 | Human immunodeficiency virus 1 infection | 13/88 | 3.23E-06 |
| hsa05132 | Salmonella infection | 13/88 | 3.3E-06 |
| hsa04010 | MAPK signaling pathway | 15/88 | 3.89E-06 |
| hsa05222 | Small cell lung cancer | 9/88 | 4.13E-06 |
| hsa05166 | Human T-cell leukemia virus 1 infection | 13/88 | 4.13E-06 |
| hsa04657 | IL-17 signaling pathway | 9/88 | 4.73E-06 |
| hsa04926 | Relaxin signaling pathway | 10/88 | 7.73E-06 |
| hsa04510 | Focal adhesion | 12/88 | 1E-05 |
| hsa01521 | EGFR tyrosine kinase inhibitor resistance | 8/88 | 1.21E-05 |
| hsa05164 | Influenza A | 11/88 | 1.29E-05 |
| hsa05145 | Toxoplasmosis | 9/88 | 1.82E-05 |
| hsa04012 | ErbB signaling pathway | 8/88 | 1.96E-05 |
| hsa04218 | Cellular senescence | 10/88 | 3.7E-05 |
| hsa05223 | Non-small cell lung cancer | 7/88 | 4.32E-05 |
| hsa04064 | NF-kappa B signaling pathway | 8/88 | 8.08E-05 |
| hsa04625 | C-type lectin receptor signaling pathway | 8/88 | 8.08E-05 |
| hsa05133 | Pertussis | 7/88 | 8.3E-05 |
| hsa05220 | Chronic myeloid leukemia | 7/88 | 8.3E-05 |
| hsa04659 | Th17 cell differentiation | 8/88 | 9.32E-05 |
| hsa04931 | Insulin resistance | 8/88 | 9.76E-05 |
| hsa05206 | MicroRNAs in cancer | 13/88 | 0.000127 |
| hsa05226 | Gastric cancer | 9/88 | 0.000145 |
| hsa04932 | Non-alcoholic fatty liver disease | 9/88 | 0.000149 |
| hsa04370 | VEGF signaling pathway | 6/88 | 0.00017 |
| hsa05010 | Alzheimer disease | 14/88 | 0.00017 |
| hsa05130 | Pathogenic Escherichia coli infection | 10/88 | 0.000172 |
| hsa05416 | Viral myocarditis | 6/88 | 0.000179 |
| hsa05143 | African trypanosomiasis | 5/88 | 0.00018 |
| hsa05235 | PD-L1 expression and PD-1 checkpoint pathway in cancer | 7/88 | 0.000188 |
| hsa04080 | Neuroactive ligand-receptor interaction | 13/88 | 0.000288 |
| hsa05221 | Acute myeloid leukemia | 6/88 | 0.000312 |
| hsa04068 | FoxO signaling pathway | 8/88 | 0.000313 |
| hsa05120 | Epithelial cell signaling in Helicobacter pylori infection | 6/88 | 0.00038 |
| hsa05230 | Central carbon metabolism in cancer | 6/88 | 0.00038 |
| hsa05142 | Chagas disease | 7/88 | 0.000402 |
| hsa04910 | Insulin signaling pathway | 8/88 | 0.000402 |
| hsa04620 | Toll-like receptor signaling pathway | 7/88 | 0.000439 |
| hsa04660 | T cell receptor signaling pathway | 7/88 | 0.000439 |
| hsa05152 | Tuberculosis | 9/88 | 0.000469 |
| hsa00980 | Metabolism of xenobiotics by cytochrome P450 | 6/88 | 0.000549 |
| hsa04014 | Ras signaling pathway | 10/88 | 0.000652 |
| hsa04725 | Cholinergic synapse | 7/88 | 0.000695 |
| hsa05165 | Human papillomavirus infection | 12/88 | 0.000728 |
| hsa04662 | B cell receptor signaling pathway | 6/88 | 0.000777 |
| hsa05204 | Chemical carcinogenesis | 6/88 | 0.000777 |
| hsa04071 | Sphingolipid signaling pathway | 7/88 | 0.000892 |
| hsa04722 | Neurotrophin signaling pathway | 7/88 | 0.000892 |
| hsa04020 | Calcium signaling pathway | 9/88 | 0.000945 |
| hsa04110 | Cell cycle | 7/88 | 0.001118 |
| hsa05135 | Yersinia infection | 7/88 | 0.001469 |
| hsa04024 | cAMP signaling pathway | 9/88 | 0.00154 |
| hsa04728 | Dopaminergic synapse | 7/88 | 0.001569 |
| hsa05216 | Thyroid cancer | 4/88 | 0.001809 |
| hsa05231 | Choline metabolism in cancer | 6/88 | 0.001809 |
| hsa05146 | Amoebiasis | 6/88 | 0.002208 |
| hsa04920 | Adipocytokine signaling pathway | 5/88 | 0.002357 |
| hsa04622 | RIG-I-like receptor signaling pathway | 5/88 | 0.002487 |
| hsa04928 | Parathyroid hormone synthesis, secretion and action | 6/88 | 0.002608 |
| hsa05202 | Transcriptional misregulation in cancer | 8/88 | 0.002872 |
| hsa05214 | Glioma | 5/88 | 0.003282 |
| hsa04921 | Oxytocin signaling pathway | 7/88 | 0.003502 |
| hsa04930 | Type II diabetes mellitus | 4/88 | 0.003734 |
| hsa04726 | Serotonergic synapse | 6/88 | 0.003759 |
| hsa04935 | Growth hormone synthesis, secretion and action | 6/88 | 0.004429 |
| hsa05144 | Malaria | 4/88 | 0.004933 |
| hsa04380 | Osteoclast differentiation | 6/88 | 0.006267 |
| hsa04658 | Th1 and Th2 cell differentiation | 5/88 | 0.007493 |
| hsa04912 | GnRH signaling pathway | 5/88 | 0.00777 |
| hsa04621 | NOD-like receptor signaling pathway | 7/88 | 0.00802 |
| hsa00052 | Galactose metabolism | 3/88 | 0.010069 |
| hsa01523 | Antifolate resistance | 3/88 | 0.010069 |
| hsa05131 | Shigellosis | 8/88 | 0.01055 |
| hsa04623 | Cytosolic DNA-sensing pathway | 4/88 | 0.010551 |
| hsa04664 | Fc epsilon RI signaling pathway | 4/88 | 0.013707 |
| hsa05203 | Viral carcinogenesis | 7/88 | 0.014437 |
| hsa04934 | Cushing syndrome | 6/88 | 0.014437 |
| hsa00982 | Drug metabolism - cytochrome P450 | 4/88 | 0.015499 |
| hsa05218 | Melanoma | 4/88 | 0.016122 |
| hsa04310 | Wnt signaling pathway | 6/88 | 0.01631 |
| hsa04630 | JAK-STAT signaling pathway | 6/88 | 0.017132 |
| hsa05140 | Leishmaniasis | 4/88 | 0.019774 |
| hsa04152 | AMPK signaling pathway | 5/88 | 0.019774 |
| hsa00983 | Drug metabolism - other enzymes | 4/88 | 0.021227 |
| hsa04540 | Gap junction | 4/88 | 0.030327 |
| hsa04140 | Autophagy - animal | 5/88 | 0.032684 |
| hsa04340 | Hedgehog signaling pathway | 3/88 | 0.032862 |
| hsa04913 | Ovarian steroidogenesis | 3/88 | 0.034344 |
| hsa05323 | Rheumatoid arthritis | 4/88 | 0.035193 |
| hsa04914 | Progesterone-mediated oocyte maturation | 4/88 | 0.042834 |
| hsa00480 | Glutathione metabolism | 3/88 | 0.044886 |
| hsa04916 | Melanogenesis | 4/88 | 0.044939 |
| hsa04015 | Rap1 signaling pathway | 6/88 | 0.049479 |
