# Supplementary material for: Systematic Investigation of the Effect of Powerful Tianma Eucommia Capsule on Ischemic Stroke Using Network Pharmacology
Source: Evid Based Complement Alternat Med. 2021 Jun 4;2021:8897313. doi: 10.1155/2021/8897313 (PMC8203382; doi:10.1155/2021/8897313)
Supplement: Supplementary Materials — All tables and molecular docking control lines can be found in supplementary materials. [file 8897313.f1.zip › 8897313.f1/Table 5.docx]

Table 5 molecular docking information

| MOL ID | MolName | Symbol | PDB ID | docking affinity (kcal/mol) |
| --- | --- | --- | --- | --- |
| control | 0G6 | PRSS1 | 1FXY | -9.63 |
| MOL000098 | quercetin | PRSS1 | 1FXY | -7.36 |
| MOL000098 | quercetin | PTGS1 | 6F2U | -6.94 |
| control | 6WN | NCOA2 | 5KRH | -6.82 |
| MOL000422 | kaempferol | PRSS1 | 1FXY | -6.56 |
| MOL000422 | kaempferol | PTGS1 | 6F2U | -6.24 |
| MOL009015 | (-)-Tabernemontanine | PTGS1 | 6F2U | -6.12 |
| MOL000098 | quercetin | F7 | 5PA9 | -6.11 |
| MOL000098 | quercetin | NCOA2 | 5KRH | -6.08 |
| MOL012286 | Betavulgarin | PTGS1 | 6F2U | -6.03 |
| MOL000422 | kaempferol | NCOA2 | 5KRH | -6.02 |
| MOL000449 | Stigmasterol | PTGS1 | 6F2U | -5.90 |
| MOL000359 | sitosterol | NCOA2 | 5KRH | -5.85 |
| MOL012286 | Betavulgarin | PRSS1 | 1FXY | -5.83 |
| MOL000422 | kaempferol | F7 | 5PA9 | -5.74 |
| MOL012286 | Betavulgarin | F7 | 5PA9 | -5.66 |
| MOL000358 | beta-sitosterol | NCOA2 | 5KRH | -5.55 |
| MOL012286 | Betavulgarin | NCOA2 | 5KRH | -5.46 |
| MOL000449 | Stigmasterol | NCOA2 | 5KRH | -5.45 |
| MOL000098 | quercetin | GABRA1 | 6HUK | -5.21 |
| MOL009015 | (-)-Tabernemontanine | NCOA2 | 5KRH | -5.10 |
| MOL000422 | kaempferol | GABRA1 | 6HUK | -5.09 |
| MOL000449 | Stigmasterol | CHRM1 | 5CVX | -5.08 |
| MOL000358 | beta-sitosterol | CHRM1 | 5CVX | -4.75 |
| MOL009015 | (-)-Tabernemontanine | GABRA1 | 6HUK | -4.60 |
| MOL000422 | kaempferol | CHRM1 | 5CVX | -4.35 |
| MOL000358 | beta-sitosterol | GABRA1 | 6HUK | -4.32 |
| MOL009031 | Cinchonan-9-al, 6'-methoxy-, (9R)- | CHRM1 | 5CVX | -4.26 |
| MOL009015 | (-)-Tabernemontanine | CHRM1 | 5CVX | -4.12 |
| MOL000449 | Stigmasterol | GABRA1 | 6HUK | -3.93 |
| MOL012286 | Betavulgarin | ESR1 | 1R5K | -5.68 |
| MOL011604 | Syringetin | ESR1 | 1R5K | -5.82 |
| MOL000098 | quercetin | RELA | 6QHL | -3.72 |
| MOL000422 | kaempferol | RELA | 6QHL | -3.52 |
| MOL002773 | beta-carotene | CTNNB1 | 4DJS | -0.64 |
| MOL000098 | quercetin | FOS | 2WT7 | -6.22 |
| MOL000449 | Stigmasterol | NCOA1 | 5MWP | -4.05 |
| MOL009027 | Cyclopamine | NR3C1 | 1P93 | -5.98 |
